# Poly(ADP-ribosyl)ation enhances HuR oligomerization and contributes to pro-inflammatory gene mRNA stabilization

**DOI:** 10.1007/s00018-020-03618-4

**Published:** 2020-08-13

**Authors:** Yueshuang Ke, Xueping Lv, Xingyue Fu, Jing Zhang, Ameer Ali Bohio, Xianlu Zeng, Wenjing Hao, Ruoxi Wang, Istvan Boldogh, Xueqing Ba

**Affiliations:** 1 The Key Laboratory of Molecular Epigenetics of the Ministry of Education, School of Life Science, Northeast Normal University, Changchun, 130024 Jilin China; 2 Institute of Genetics and Developmental Biology, Chinese Academy of Sciences, Beijing, 100101 China; 3 Institute of Biomedical Sciences, College of Life Sciences, Key Laboratory of Animal Resistance Biology of Shandong Province, Shandong Normal University, Jinan, 250014 Shandong China; 4 Department of Microbiology and Immunology, University of Texas Medical Branch at Galveston, Galveston, TX 77555 USA

**Keywords:** HuR, Inflammation, Oligomerization, PARP1, mRNA stability

## Abstract

Poly(ADP-ribosyl)ation (PARylation) is an important post-translational modification mainly catalyzed by poly-ADP-ribose polymerase 1 (PARP1). In addition to having important roles in DNA damage detection and repair, it functions in gene expression regulation, especially at the posttranscriptional level. Embryonic lethal abnormal vision-like 1/human antigen R (ELAVL/HuR), a canonical 3′ untranslated region AU-rich element-binding protein, is a crucial mRNA-stabilizing protein that protects target mRNAs from RNA-destabilizing protein- or microRNA-induced silencing complex (miRISC)-mediated degradation. Additionally, in some cases, HuR itself either promotes or suppresses translation. Here, we demonstrated that in response to inflammatory stimuli, the PARylation of HuR, mostly at the conserved D226 site, by PARP1 increased the formation of the HuR oligomer/multimer, and HuR oligomerization promoted the disassociation of miRISC and stabilized the pro-inflammatory gene mRNAs. The prevention of PARP1 activation or HuR oligomerization attenuated lipopolysaccharide-induced inflammatory gene expression and the airway recruitment of neutrophils in mouse lungs. The present study verified a novel mechanism of PARP1 and HuR PARylation in the RNA stability regulation, increasing our understanding of how PARP1 regulates gene expression.

**Electronic supplementary material:**

The online version of this article (10.1007/s00018-020-03618-4) contains supplementary material, which is available to authorized users.

## Introduction

Eukaryotes employ multiple post-transcriptional mechanisms to adjust their gene expression levels in response to external stimuli and changes in cellular physiopathology. Many proteins that regulate cell growth, differentiation and inflammation are coded by unstable mRNAs. Among these mRNAs are short-lived mRNAs, which are often characterized by the presence of cis-regulatory elements that are responsible for their degradation [1]. One class of such elements is represented by the AU-rich elements (AREs), which are mainly present in 3′ untranslated regions (3′ UTRs) and bound by RNA-binding proteins (RBPs) [2, 3]. While most ARE-BPs function as negative regulators of posttranscriptional gene expression by decreasing mRNA stability or translation, embryonic lethal abnormal vision-like/human antigen (ELAVL/Hu) proteins commonly function as positive posttranscriptional regulators of gene expression by causing increases in mRNA stability [1, 4, 5].

Four proteins belong to the Hu family. HuR (HuA) is expressed ubiquitously in all tissues, whereas HuB (or Hel-N1), HuC and HuD are usually considered neuron-specific [6, 7]. HuR has been implicated in a series of physiological and pathological processes through its post-transcriptional regulation of target genes that are involved in cell proliferation, anti-apoptosis and inflammatory responses [1, 8–13]. HuR is localized predominantly in the nucleus and shuttled into the cytoplasm using a HuR nucleocytoplasmic shuttling sequence (HNS) under various stress conditions [7, 12, 14]. HuR may compete with other RBPs or micro (mi) RNAs to bind to target mRNAs and regulate mRNA stability [15, 16]. The functional regulation of HuR is achieved through protein modifications, including phosphorylation, methylation, ubiquitination, NEDDylation and proteolytic cleavage, which may regulate its subcellular localization, interactions with other proteins or associations with target RNAs [12, 17–26].

Poly ADP-ribosylation (PARylation), an essential post-translational protein modification in cells, is catalyzed by the PARP superfamily by attaching poly ADP-ribose (PAR) to target proteins using nicotinamide adenine dinucleotide as a donor. PARP1, as the most abundant and ubiquitous member of the family, accounts for most of the cellular PARP activity [27, 28]. Increasing evidence suggests roles for the PARP superfamily in RNA metabolism, including RNA splicing, polyadenylation and nuclear export [27, 29–32]. Our previous studies showed that the mRNA stability level of a set of pro-inflammatory cytokines/chemokines (e.g., *Cxcl1*, *Cxcl2*, *Cxcl13* and *Il*-*1β*) was modulated by PARP1-HuR signaling. Inflammatory stimuli induce the PARP1 interaction with HuR and the PARylation of the latter at the D226 site, which is located in the HNS domain. PARylation endows HuR with enhanced nuclear-cytoplasmic shuttling and increases the association with target mRNAs. The PARP1 depletion or inhibition significantly destabilizes the mRNAs of cytokines/chemokines and restricts the inflammation [14]. Although PARP1’s role in HuR-mediated mRNA stability is beyond doubt, the underlying molecular mechanism still remains to be fully understood.

We proposed previously that the PARylation of D226 in the HNS domain leads to its conformational change, which facilitates the association of HuR with its partners and/or mRNA targets, resulting in the functional regulation of HuR. Hu family members interact with themselves in neuronal cells, and it is believed that these interactions may contribute to the temporal storage of ARE-containing mRNA [15, 33]. HuR forms multimers in glioma cells, which contributes to glioma cancer progression [34]. These studies compelled us to investigate whether PARP1 regulates HuR-mediated mRNA stability by influencing the oligomerization/multimerization of HuR.

In the present study, we observed that, in response to inflammatory stimulation, the self-interaction of HuR was enhanced upon HuR PARylation. The oligomerization/multimerization of HuR along the RNA substrate promotes the dissociation of the miRNA-induced silencing complex (miRISC) from the target RNA, thereby stabilizing ARE-containing mRNAs. In summary, we provide new insights into the significance of HuR oligomerization in cells subjected to inflammatory stimuli and demonstrate an essential role, as well as the underlying mechanism, of PARylation during this process.

## Materials and methods

### Cell culture, treatment, and transfection

Human Embryonic Kidney cells (HEK293) were cultured in DMEM (Invitrogen) supplemented with 10% (v/v) Fetal Bovine Serum (FBS). The dose of recombinant human TNFα (300-01A, Peprotech) was 10 ng/mL. PJ34 (P4365, Sigma, 2.5 μM), Olaparib (Ola, AZD2281, Selleckchem, 10 nM), transcription inhibitor actinomycin D (Act D, A1410, Sigma) and tanshinone group compound 15,16-dihydrotanshinone-I (DHTS, HY-N0360, MedChemExpress, 10 μM) were added directly into the culture medium. siRNAs targeting PARP1 (5′-CCAAAGGAATTCCGAGAAA-3′) and siRNA targeting HuR (5′-TGCCGTCACCAATGTGAAAGT-3′) were used at 100 nM. Lipofectamine 2000 (Invitrogen) was used for transfection of siRNAs and plasmids.

### In situ chemical crosslinking analysis

HEK293 cells were suspended in PBS and incubated with or without 1 mM disuccinimidyl suberate (DSS) (21555, Thermo Scientific) for 30 min at room temperature, then quenched by adding quenching buffer to a final concentration of 20 mM Tris at room temperature for 15 min [33]. After the treatment, the cells were lysed with radio immunoprecipitation assay (RIPA) buffer, and the extracts were separated by electrophoresis on 10% SDS polyacrylamide gels, followed by western blotting with anti-HuR antibody.

### Duolink (proximity ligation assay, PLA)

The proximity ligation assay (PLA) (Dolin In Situ Detection Reagents Red, DUO92008, sigma) was performed following the manufacturer’s instructions. Cells were growing on slides, fixed with paraformaldehyde 4% and blocked for 1 h, then incubated with the primary antibodies against GFP or HA and FLAG overnight at 4 °C. IgG was used as a negative control. Each dot corresponds to a close interaction. A pair of oligonucleotide-labeled secondary antibodies (PLA probes) binds to the primary antibodies, and generates a signal only when the two probes are in proximity. Images were acquired on a confocal microscope (LSM880, ZEISS, Germany).

### Constructs

Plasmids Flag-HuR, GST and GST-HuR were kindly provided by Dr. Myriam Gorospe (Laboratory of Cellular and Molecular Biology; National Institute on Aging, National Institutes of Health, USA). Plasmid GFP-HuR was provided by Dr. Imed-Eddine Gallouzi (Department of Biochemistry, Division of Critical Care, McGill University Health Center, McGill University, Montreal, Quebec H3G 146, Canada). Mir51 primary precursor plasmid was obtained from Dr. Yu Zhang (Key Laboratory of Molecular Epigenetics of Ministry of Education, School of Life Sciences, Northeast Normal University).

His-HuR and HA-HuR were constructed by subcloning HuR amplicons into His (PET30a) tagging and pCDNA3.1 vectors. To construct Flag-Ago2 plasmid, Ago2 was amplified by PCR using human cDNA as the template and then cloned into the vector pCMV-N-Flag. To construct Flag-mHuR and GFP-mHuR plasmids, murine HuR were amplified by PCR using mouse cDNA as the template and then cloned into the vector pCMV-N-Flag and pGFP-C1. The domain mutations GST-HuR-RRM1, GST-HuR-RRM2, GST-HuR-HNS, GST-HuR-RRM3, GST-HuR-△RRM1, GST-HuR-∆RRM1 + RRM2, GST-HuR-∆HNS + RRM3 and GST-HuR-△RRM3 were developed from GST-HuR. A Fast Mutagenesis System kit (FM111, TRANS, Beijing, China) was used to produce W261E and D226A site mutations in background of GST-HuR, His-HuR, Flag-HuR and GFP-HuR.

### Cell fractionation

Whole cell lysis buffer, as well as cytosolic and nuclear fractionations were performed as described [14]. Briefly, Whole-cell lysates, prepared in radio immunoprecipitation assay (RIPA) buffer on ice for 30 min. Lysates were centrifuged and the supernatants were the whole cell extract (WE). Cytoplasm and nuclear fractions were prepared by using the CelLytic NuCLEAR Extraction Kit (Sigma, NXTRACT, Saint Louis, MO). Cells were lysed with Cytosolic Lysis Buffer for 20 min, lysates were centrifuged (11,000×*g*, 1 min, 4 °C), and supernatants were collected as cytosolic extracts (CE). The pellets were washed twice with Cytosolic Lysis Buffer and lysed with Extraction Buffer. Nuclear lysates were clarified by centrifugation (21,000×*g*, 5 min, 4 °C), and the supernatants were collected (nuclear extract, NE).

### Immunoblotting and immunoprecipitation

Cells were cultured and stimulated as described above and lysed. Then, protein from each sample was resolved by SDS-PAGE. After proteins were transferred to nitrocellulose membrane, the membranes were washed with TBST (20 mM Tris base, 500 mM NaCl, 0.1% Tween-20, pH 7.5), blocked with 5% non-fat dry milk. Blots signals were detected by using ECL plus, a chemiluminescent detection system (180-5001, Tanon, Shanghai, China), after incubated with primary antibody overnight and then horseradish peroxidase-conjugated secondary antibody for 1 h. Primary antibodies used were: anti-HuR (3A2, sc-5261), anti-Histone H1 (AE-4, sc-8030) all from Santa Cruz Biotechnology (Santa Cruz, CA). Anti-β-tubulin (HC101), anti-β-actin (HC201), anti-GFP (HT801) and anti-His (HT501) were purchased from TRANS (BeiJing, China). Monoclonal antibody against polyADP-ribose (PAR) (ALX-804-220) was from Alexis (San Diego, CA). Monoclonal antibody against FLAG (F1804) was from Sigma (Saint Louis, Missouri). The anti-FLAG rabbit polyclonal antibody (20543-1-AP) was from ProteinTech (Wuhan, China). Anti-Argonaute 2 antibody (Ago2, C34C6, 2897) was from Cell Signaling Technology (Danvers, MA). For co-Immunoprecipitation (co-IP) analysis, CE, NE, and WE were incubated with antibodies recognizing HuR, or FLAG prebound to protein G Agarose/Salmon. Mouse IgG1 (Santa Cruz, CA) were used in control IP reactions. Protein–protein interactions were studied by Western blot analysis of IP samples. The western blot signal was detected with ECL Plus reagents (S6010, US EVERBRIGHT). The relative band intensities were quantified by densitometry using the ImageJ software (1.41 V, US National Institutes of Health).

### Purification of fused proteins

Overnight cultures of *E. coli* BL21 transformed with plasmids expressing HuR or its mutants were diluted at 1:100 with the LB medium. Then, cultures were induced with IPTG (1 mM) and grown at 37 °C for 2–3 h. Cells were spun down and lysed in buffer containing 20 mM Hepes, pH 7.5, 120 mM NaCl, 5% glycerol, and protease inhibitor cocktail (Roche). The lysate was centrifuged at 12000 g for 25 min at 4 °C. The supernatant was incubated with glutathione Sepharose 4B (GE Healthcare Life Science, Uppsala, Sweden) or Ni–NTA Agarose beads (Qiagen). The GST-fused proteins were purified and eluted in elution buffer (50 mM Tris–HCl (pH>8.0), 100 mM KCl and 40 mM glutathione).

### In vitro PARylation assay

A GST-fused protein in vitro PARylation assay was set up by modifying the method provided by the HT Universal Chemiluminescent PARP Assay kit (Trevigen, Gaithersburg, MD, USA) [14]. Briefly, GST and GST-fused proteins immobilized on glutathione Sepharose 4B were incubated with recombinant PARP enzyme and PARP cocktail at room temperature for 1 h. After three washes with Nonidet P-40 lysis buffer, the bound proteins were analysed by western blotting.

### RNA-EMSA

Interactions of HuR and the mutants with target RNAs were analyzed by electrophoretic mobility shift assays (20158, Thermo Fisher Scientific, Waltham, MA USA). Briefly, proteins (with or without PARylation) were dissolved in the EMSA interaction buffer (3 mM MgCl_2_, 40 mM KCl, 5% glycerol, 2 mM DTT, 2 μg tRNA) and incubated with 5 nM of 5′ biotin-labeled RNA oligos for 40 min at room temperature. The reaction mix was then loaded on to a 6% acrylamide native gel. RNA oligo probes utilized in the present study included: AU-rich RNA oligo: 5′-AUUUAUUUAUUUAUUUAUUUAUUUA-3′; Cxcl2 RNA oligo: 5′-CUAUGUAUUUAUUUAUUUAUUAAUU-3′.

### Purification of miRISC

MiRISC complexes were purified from HEK293 cells (grown in 10-cm dish) doubly transfected with mir51 primary precursor (pri-miRNA) or let-7a miRNA and Flag-tagged Ago2 protein. 48 h after transfection, cells were lysed with Cytosolic Lysis Buffer. After centrifugation at 11,000×*g*, the supernatant was used for IP purification with 2 μg anti-FLAG antibody (Sigma). The miRISC complex was eluted with FLAG peptide (F3290, Sigma), following the manufacturer’s protocol.

### In vitro transcription

The in vitro transcription was performed by MEGAshortscript™ T7 Transcription Kit (AM1354, Thermo Scientific). Briefly, for each target RNAs (Supplementary Table 1 and Supplementary Table 2), transcription reactions (20μL), containing 2μL transcription buffer, 150 mM each ATP, CTP, GTP and UTP, 2.5 mM DTT, and 2μL of T7 Enzyme Mix, were performed at 37 °C for 2 h with 50 nM of template (Supplementary Table 1). The target RNA (Supplementary Table 2) was recovered by chloroform extraction and alcohol precipitation following the manufacturer’s instructions.

### Target RNA cleavage assay

The reactions (10 μL), containing 1 μL cleavage buffer (20 mM Hepes–KOH, pH 7.4, 150 mM KCl, 2 mM MgCl_2_, 0.01% Triton X-100, 5% glycerol, 1 mM DTT), 0.1 mg yeast tRNA, purified miRISC, 0.1 nM target RNA (Supplementary Table 2) and increasing amounts of indicated HuR proteins, were incubated for 30 min at 30 °C. Reactions were stopped by adding urea stop dye (8 M urea, 50 mM EDTA, 0.04% bromophenol blue, 0.04% xylene cyanol), followed by heating at 95 °C for 2 min. The products were analyzed by 8 M urea PAGE.

### IP of RNP complexes

For IP of RNP complexes, whole cell lysate were pre-cleared and immunoprecipitated by using protein G Agarose/Salmon coated with anti-HuR, FLAG antibodies or alternatively, the same amount of IgG1 (2 μg). After the beads were washed with NT2 buffer (50 mM Tris–HCl at pH 7.5, 150 mM NaCl, 1 mM MgCl_2_ and 0.05% NP-40), the beads-antibody- protein/mRNA-bound complexes for each sample were further assessed by reverse transcription (RT). The mRNA was isolated by using an RNA sample total RNA kit (DP419, TIANGEN, China). The level of mRNA was measured by quantitative RT-PCR, using β-actin the internal control to normalize and monitor the variations of gene expression. To determine the stability of the mRNA in the HEK293 cells, we used CXCL2 primers: forward: 5′-CAAACCGAAGTCATAGCC-3′, reverse: 5′-GAACAGCCACCAATAAGC-3′. CXCL1 primers: forward: 5′-TCTCTCTTTCCTCTTCTGTTCCTA -3′, reverse: 5′-CATCCCCCATAGTTAAGAAAATCATC-3′. TNFα primers: forward: 5′-TCAGCTTGAGGGTTTGCTAC-3′, reverse: 5′-TGCACTTTGGAGTGATCGG-3′. β-actin primers: forward: 5′-CTCCATCCTGGCCTCGCTGT-3′, reverse: 5′-GCTGTCACCTTCACCGTTCC-3′.

### Stability of mRNA

To measure the stability of the inflammatory mediator mRNA, a classical approach is applied. Endogenous HuR in HEK293 cells was silenced using siRNA targeting a distinguished sequence of human HuR, and then WT mHuR, W261E and D226A mHuR expressional plasmids were transfected. The difficult treated cells were exposed to TNFα for 0.5 h, then transcription inhibitor Act D was added to the medium with or without the maintenance of TNFα (± Ola or DHTS) for 0, 2 and 4 h. RNA was isolated with Trizol (Invitrogen) and its concentration and integrity were determined. PCRs were performed using Applied Biosystems thermocycler by the ∆∆Ct method, using β-actin as reference gene.

### Mouse work

Six- to eight-week-old female C57BL/6 mice (20–25 g) were purchased from Jilin University (Changchun, Jilin, China). Mice were housed in a specific pathogen-free facility at NENU (Changchun, Jilin, China) and allowed unlimited access to sterilized feed and water. They were maintained at 23 °C ± 1 °C and kept under a 12-h light/dark cycle. All experiments were conducted in accordance with the Chinese Council on Animal Care Guidelines. Mice were challenged with LPS (1 mg/kg) using the intranasal route, with or without an intraperitoneal pretreatment of Ola (10 mg/kg) or DHTS (5 mg/kg) 30 min prior to the LPS challenge. Mice lungs were harvested and homogenates were prepared. To determine the level of the mRNA in the mice lung, primers were applied: mCxcl2: forward: 5′-TCAATGCCTGAAGACCC-3, reverse: 5′-TGGTTCTTCCGTTGAGG-3′; mβ-actin: forward: 5′-AACAGTCCGCCTAGAAGCAC-3′, reverse: 5′-CGATGACATCCGTAAAGACC-3′. mTnf-α: forward: 5′-AGA CCC TCA CAC TCA GAT CA-3′, reverse: 5′-TCT TTG AGA TCC ATG CCG TTG-3′.

### Evaluation of airway inflammation

Evaluation of mouse lung airway inflammation was performed as described [35]. Briefly, tracheae were cannulated, and lungs were lavaged by two instillations of ice-cold PBS after LPS challenge for 16 h. Bronchoalveolar lavage fluid (BALF) samples were centrifuged and the supernatants were stored at -80 °C for further analysis. The pellet included total cells, for which cell counts in the BALF were determined from an aliquot of the cell suspension using a hemocytometer. Differential cell counts were performed on centrifuge preparations. Cells were stained with modified Wright–Giemsa using HEMA-TEK 2000 Slide Stainer (Protocol) for differential cell counts. Randomly selected fields were photographed using an OLYMPUS Microscope System BX53P microscope with a built-in digital.

### Statistical analysis

All experiments were performed at least three times for each determination. Data were expressed as means ± standard deviations (*n* ≥ 3) and analyzed by one-way or two-way analyses of variance. The level of significance was accepted at *p < 0.05, **p < 0.01 and ***p < 0.001.

## Results

### Inflammatory stimulation promotes HuR oligomerization in cells

**Fig. 1:**
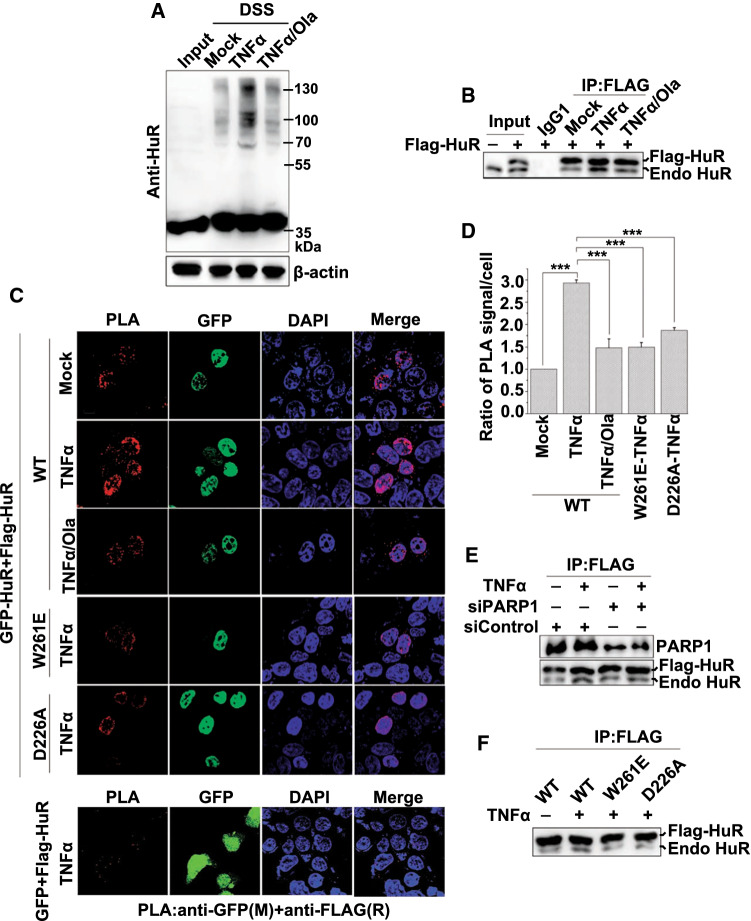
PARP1 is crucial for inflammatory stimulation-induced HuR oligomerization in cells. **a** PARylation is required for the stimulation-dependent increase in HuR oligomerization shown by in situ chemical crosslinking. HEK293 cells were either exposed to TNFα (± Ola) for 1 h or not, and then the cells receiving different treatments were suspended independently in PBS and incubated with or without 1 mM DSS. After quenching the reaction using a final concentration of 20 mM Tris at room temperature for 15 min, the extracts were lysed with RIPA buffer, followed by western blotting with an anti-HuR antibody. **b** HuR undergoes self-associate in cells responding to inflammatory stimuli. HEK293 cells were transfected with the Flag-HuR plasmid and treated with or without TNFα (± Ola). Cells extracts were immunoprecipitated with control IgG1 or an anti-FLAG antibody, and the precipitants were analyzed by western blotting with an anti-HuR antibody. Endo HuR, endogenous HuR. **c**, **d** Oligomerization of HuR and the mutants were analyzed using a PLA. GFP-HuR together with Flag-HuR or mutated plasmids, as indicated, was transfected into cells. The cells were mock-treated or TNFα-exposed (± Ola) for 1 h, and then subjected to in vivo PLA assays with indicated antibodies. Scale bar, 10 μm (**c**). The quantitative results (**d**) are represented from three independent experiments. ***p < 0.001. **e** PARP1 knockdown impairs HuR oligomerization. HEK293 cells were transfected with PARP1 siRNA or a control, and then, the Flag-HuR expression plasmid was transfected independently. After 48 h, the differently treated cells were exposed to TNFα for 1 h, and then, immunoprecipitates were prepared using a FLAG antibody. They were subjected to western blotting to detect the interaction of Flag-HuR with the endogenous HuR. Endo HuR, endogenous HuR. **f** The HuR D226 mutation decreases its oligomerization in response to TNFα exposure. HEK 293 cells were transfected independently with wild-type (WT) Flag-HuR, W261E and D226A mutant plasmids, and then either challenged with TNFα or not for 1 h. Immunoprecipitates were prepared using control IgG1 or an anti-FLAG antibody and then subjected to western blotting to detect the interaction of Flag-HuR with the endogenous HuR. Endo HuR, endogenous HuR

To examine the occurrence of HuR oligomerization in response to inflammatory stimulation, we performed an in situ chemical crosslinking analysis [33]. Cells were challenged with TNFα for 1 h, treated with the amine-specific chemical crosslinking reagent disuccinimidyl suberate (DSS), and then, the HuR contents under non-denaturing and non-reducing conditions were analyzed by western blotting using an anti-HuR antibody (Ab). In addition to the monomeric HuR located at ~ 36 kDa, three specific crosslinked complexes were detected using the crosslinkers. The complexes had molecular masses of ~ 70, 100 and 130 kDa, and were likely to be the dimer, trimer and tetramer of HuR, respectively. Furthermore, an overt increase in the level of HuR oligomerization was observed in TNFα-treated cells (Fig. 1a and Figure S1A). To rule out the possibility that other cell proteins cross-linked with HuR under DSS-treatment conditions and to confirm the formation of the HuR oligomer in response to inflammatory stimulation, Flag-tagged HuR (Flag-HuR) was expressed in HEK293 cells, and then, the cell extracts were subjected to immunoprecipitation using anti-FLAG Ab. The TNFα exposure increased the level of endogenous HuR pulled-down by epitope-tagged HuR compared with in untreated cells (Fig. 1b and Figure S1B). Additionally, we transfected GFP-HuR and Flag-HuR plasmids into cells, and then, the cell extracts were subjected to immunoprecipitation using anti-FLAG Ab. The amounts of pulled-down GFP-HuR and endogenous HuR also increased in response to TNFα exposure (Figure S1C). To further validate the direct self-interaction of HuR, a proximity ligation assay (PLA) was performed. We transfected GFP or GFP-HuR together with Flag-HuR into cells, and by detecting the annealing and amplification of a pair of PLA probes that recognized GFP and FLAG, respectively, we observed a substantial increase in the Flag-HuR and GFP-HuR interactions, but not the Flag-HuR and GFP interactions, in TNFα-treated cells (Fig. 1c and d). In addition, we performed the PLA assay by transfecting HA-HuR and Flag-HuR into cells, and TNFα-induced interactions between HA-HuR and Flag-HuR were observed (Figure S1D). Thus, our data indicated that HuR oligomerization increased in cells exposed to inflammatory stimuli.

### PARP1 activity is required for enhanced HuR oligomerization upon inflammatory stimulation

Our previous work showed that PARP1 covalently PARylates HuR and influences its function under lipopolysaccharide (LPS) stimulation [14]. This led us to investigate whether PARP1 regulates the formation of the HuR oligomer. Thus, an in situ chemical crosslinking analysis was performed, and cross-linked complexes of the HuR oligomer in TNFα-treated cells markedly increased. This was blocked by the addition of PARP inhibitor Olaparib (Ola) (Fig. 1a and Figure S1A). In addition, the amount of the endogenous HuR in the complexes co-immunoprecipitated along with Flag-HuR from cells treated with Ola was examined. The TNFα treatment-induced increase in the level of endogenous HuR in Flag/GFP-HuR-immunoprecipitated complex was abolished by Ola (Fig. 1b, Figure S1B and Figure S1E). Moreover, PLA results showed that the Ola treatment markedly reduced the HuR self-interaction level (Fig. 1c and d). To determine the specific role of PARP1 in the oligomerization of HuR, we used siRNA that targeted PARP1. The protein co-immunoprecipitation assay revealed that the oligomerization of HuR in PARP1-interfered cells decreased compared with in control siRNA-transfected cells (Fig. 1e and Figure S1F). The D226 site of HuR is a major site for the PARylation of HuR [14]; and the oligomerization of HuR may be mediated by Trp261 (W261), a surface-exposed residue conserved in the RNA recognition motif (RRM)3 of HuR paralogues and orthologues [36, 37]. The roles of these two crucial sites in HuR oligomerization were further investigated. Our PLA and co-immunoprecipitation results confirmed that the W261E mutant had a significant loss in the capability to closely self-interact, and importantly, the D226A mutant, which was not PARylated upon inflammatory stimulation, showed a pattern similar to that of the W261E mutant (Fig. 1c, d, f and Figure S1G).

## The D226 site of HuR is crucial for the PARP1-induced enhancement of HuR self-interactions

Both PARP1 and HuR are targets of caspases and may undergo cleavage under lethal stress conditions [26, 38]. Additionally, D226 of HuR is recognized by caspases (e.g., caspase-3 and -7) [26]. Thus, the whole-cell lysate under TNFα stimulation was applied, along with a molecular weight standard, and HuR and PARP1 did not undergo caspase-mediated cleavage under inflammatory conditions (Figure S2A).

**Fig. 2:**
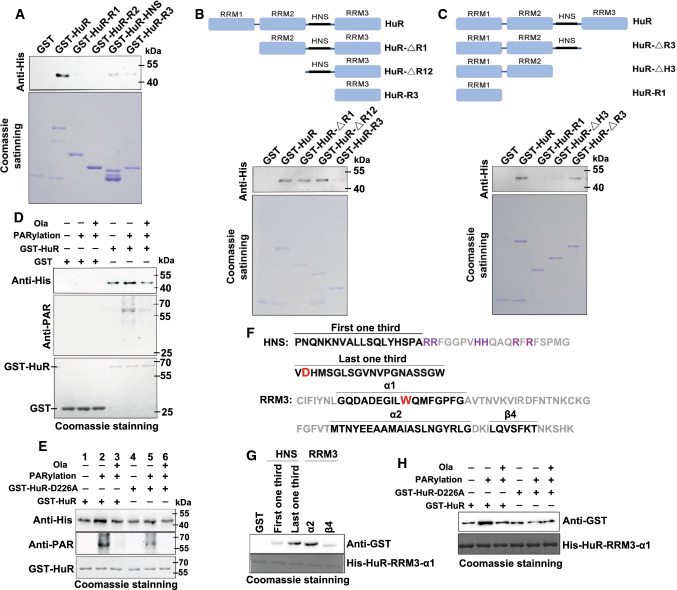
PARylation at D226 promotes the formation of HuR oligomerization. **a–c** Hinge and RRM3 domains are necessary for HuR oligomerization. GST and GST-HuR, as well as the domain (**a**) and truncated (**b**, **c**) mutants were incubated with equal amounts of His-HuR. Levels of pulled-down His-HuR were detected by western blotting. **d**, **e** PARylation of HuR at D226 increased its oligomerization. An in vitro PARylation assay was performed using purified GST, GST-HuR (**d**) and D226 mutants (**e**) in the presence or absence of recombinant PARP1 enzyme. Then, PARylated GST, GST-HuR (**d**) and D226 mutants (**e**) were incubated with His-HuR, and subjected to western blotting with a His antibody. **f** Amino-acid sequence information of HNS and RRM3 from HuR. Helices α1 and α2 and beta sheet β4 of RRM3 together with the first third and the last third of HNS are indicated. Basic amino acids located in the second third of HNS and presenting NLS are highlighted in purple. Crucial sites for PARylation (D226) and oligomerization (W261) are enlarged and highlighted in red. **g** The interactions between the α1 helix of HuR RRM3 and other modules. GST, GST-tagged α2 and β4 of RRM3, as well as the first, third and the last third of HNS, were purified and eluted, followed by incubation with His-HuR-RRM3-α1. The interactions between the RRM3 α1 helix and other modules were investigated using western blotting with a GST antibody. **h** The D226 PARylation provides favorable interface for HuR oligomerization. An in vitro PARylation assay was performed using purified the full-length HuR and D226 mutant. Then, PARylated HuR and D226 mutation were incubated with His-HuR-RRM3-α1, and subjected to western blotting by using a GST antibody

Next, domain(s) that mediate HuR self-interaction were verified. Recombinant GST- and His-tagged HuR were induced and purified for pull-down experiments. Sepharose 4B beads coated with different concentrations of GST or GST-HuR were incubated with the same amount of His-HuR. GST-HuR (but not GST) markedly interacted with His-HuR (Figure S2B), and similar results were obtained by incubating His-HuR-coated beads with GST or GST-HuR (Figure S2C). The interactions between differently tagged HuR occurred in a dose-dependent manner. Previous studies utilizing yeast two-hybrid, nuclear magnetic resonance and molecular dynamics simulation implicated roles for the HNS and RRM3 domains in homo- and heterogeneous helical hydrophobic interactions between ELAVL family members [16, 33, 36, 37, 39]. Here, by constructing the HuR domain and truncated mutant plasmids, our recombinant protein pull-down experiments confirmed that the oligomerization of HuR depended on the HNS and RRM3 domains (Fig. 2a–c).

Furthermore, we assessed the impact of PARylation on HuR dimerization utilized an in vitro PARylation system [14]. GST or GST-HuR was purified and either subjected to PARylation or not, followed by incubation with His-HuR. Increases in the PARylation of GST-HuR and its dimerization with His-HuR were observed compared with untreated GST-HuR; however, the dimerization was significantly lowered to the basal level by the addition of Ola (Fig. 2d and Figure S2D). The addition of another PARP inhibitor PJ34 or poly(ADP-ribose) glycohydrolase (PARG), the enzyme that removes ADP-ribose units from target proteins, also reduced the enhancement of HuR dimerization (Figure S2E). To address the importance of PARylation of D226 site for HuR oligomerization, D226A mutant of GST-HuR was purified and subjected to PARylation or not. D226A mutation significantly inhibits the increase in HuR dimerzation caused by PARylation (Fig. 2e and Figure S2F, compared the lanes 4, 5 to lanes 1,2).

The RRM domain in the Hu family members is composed of two α-helices packed against an anti-parallel β-sheet with a β1-α1-β2-β3-α2-β4 topology [40]. Three conserved short sequences in RRM3 play critical roles in ELAVL–ELAVL interactions. Two of these short sequences, one of which is the crucial W261, are located in the α1 helix (Fig. 2f), and the remaining sequence is located in the α2 helix. Moreover, the last third of HNS is also indispensable for ELAV multimerization [41]. D226, the site that undergoes PARylation in HuR, is located at the boundary between the second third and the last third of the HNS (Fig. 2f). This characteristic further suggests that D226 PARylation is highly relevant to HuR’s oligomerization. We speculate that, to achieve oligomerization/multimerization, a parallel and interlaced interaction between HNS and RRM3 domains occurs. HuR’s α1 helix may mediate HuR’s self-interaction by interacting with the last third of the HNS and with the α2 helix of the RRM3 in another HuR. To test this hypothesis, we constructed GST-tagged first third and the last third of HNS, as well as GST-tagged α2 and β4 units of RRM3, and then used the His-tagged α1 helix of RRM3 to perform pull-down experiments (Fig. 2f, Figure S2G and Supplementary Table S3). His-HuR-RRM3-α1 significantly interacted with the α2 helix and the last third of HNS, but not with the first third of HNS or β4 unit (Fig. 2g). To further support our hypothesis that PARylation of D226 causes conformation change of HuR, leads to the last one-third of HNS as well as α2 helix more exposed for α1 helix of RRM3, the WT HuR together with D226A mutant were utilized to perform PARylation or not, followed by incubation with His-HuR-RRM3-α1. Results showed PARylation of the WT HuR, but not D226A mutant, increased the interaction with His-HuR-RRM3-α1 (Fig. 2h and Figure S2H). This result implied that D226 PARylation functions as a switch and accommodates the last third of HNS together with RRM3 as a favorable interface for HuR oligomerization/multimerization.

### PARylation enhances HuR oligomerization in both the cytoplasm and nucleus

**Fig. 3:**
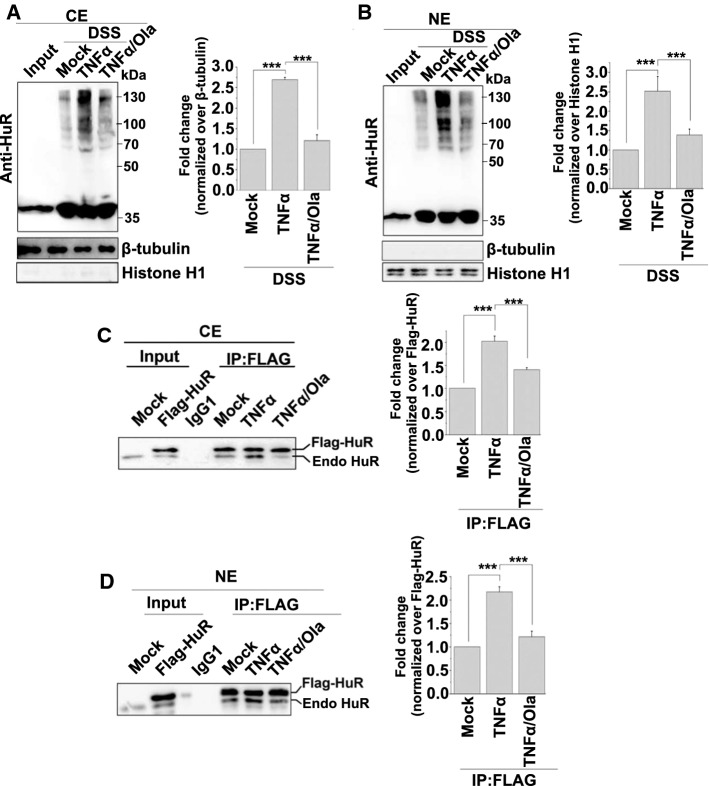
PARP1 increased HuR oligomerization in both the cytoplasm and nucleus. **a**, **b** HuR oligomerization was analyzed using an in situ chemical crosslinking analysis. HEK293 cells either exposed to TNFα (± Ola) for 1 h or not, and then the differently treated cells were suspended in PBS and incubated with or without 1 mM DSS. After quenching the reaction using a final concentration of 20 mM Tris at room temperature for 15 min, the cells was lysed using a CE (**a**) or NE (**b**) buffer and then analyzed using western blotting with an anti-HuR antibody. The oligomerization of HuR was quantified by analysis of band densitometry using the ImageJ software (right panel). ***p < 0.001; *n*=3. **c**, **d** HuR oligomerization was analyzed using an immunoprecipitation assay. Flag-HuR transfected cells were mock-treated or TNFα-exposed (± Ola) for 1 h. CE (**c**) or NE (**d**) were prepared and immunoprecipitates were obtained using antibodies recognizing FLAG. The association of Flag-HuR with endogenous HuR was detected by western blotting. The amount of co-precipitated endogenous HuR was quantified by analysis of band densitometry using the ImageJ software (right panel). ***p < 0.001; *n* = 3. Endo HuR, endogenous HuR

HuR is predominantly localized in the nucleus but undergoes cytoplasmic translocation under various cellular and stress conditions to stabilize its targets and promote translation. The PLA results revealed that the signals for HuR interactions were located in both the cytoplasm and nucleus (Fig. 1c). To confirm this observation, we further prepared cytosolic extracts (CEs) and nuclear extracts (NEs) to perform in situ chemical crosslinking analyses. Differently treated cells were subjected to DSS crosslinking, and the CEs or NEs were analyzed by western blotting using an anti-HuR Ab. After TNFα exposure and PARP1 activation, the oligomerization of HuR was enhanced in both compartments, and this was eliminated by Ola (Fig. 3a and b).

Then, HEK293 cells were transfected with the Flag-HuR plasmid and subjected to different treatments. CEs and NEs were prepared, and immunoprecipitation assays were performed by using an anti-FLAG Ab. The TNFα exposure increased the level of endogenous HuR pulled-down by epitope-tagged HuR compared with untreated cells from both in CEs and NEs, and this reaction was significant mitigated by a PARP1 inhibitor (Fig. 3c and d). The combined data suggested that the PARylation of HuR in the nucleus promotes its oligomerization, and this oligomerization was sustained until HuR was shuttled into the cytoplasm.

### PARylation promotes reciprocity between HuR’s substrate association and oligomerization

**Fig. 4:**
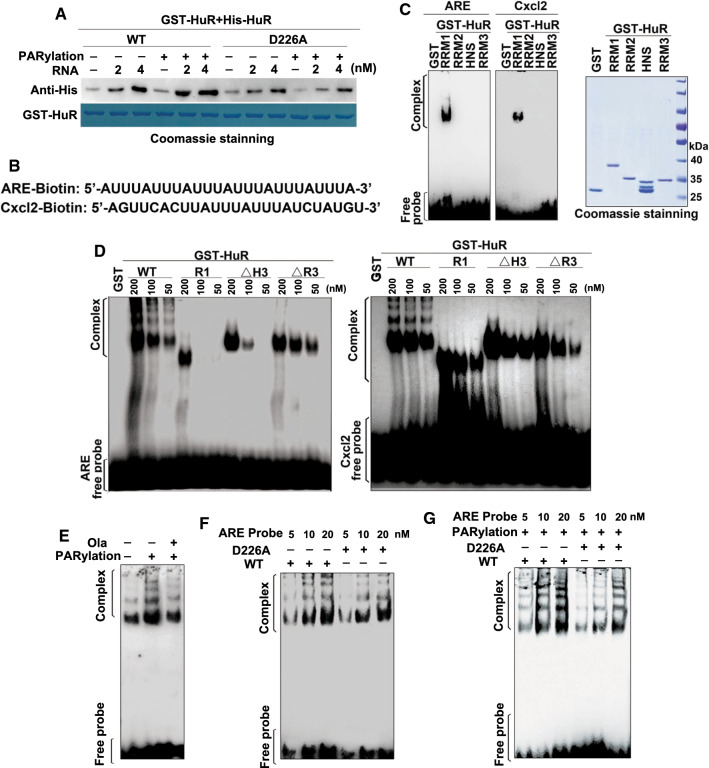
PARylation promotes HuR oligomerization along target RNA. **a** PARylation increased HuR’s self-interaction in the presence of RNA. Purified GST-HuR and the D226 mutant were either subjected to in vitro PARylation or not, and then incubated with the His-HuR protein together at different RNA concentrations. They were then subjected to western blotting using an anti-His antibody. **b** Illustration of the biotin-labeled tandem ARE repeat and Cxcl2-ARE RNA oligos. **c** The RRM1 domain mediates HuR interactions with the target mRNA. GST-HuR domains were purified and eluted, and then incubated with the biotin-labeled tandem ARE repeat and Cxcl2-ARE RNA oligos as indicated (**b**). The gel retardation assay was performed to detect the binding of proteins to probes. **d** The oligomerization potentials of HuR and its mutants were determined by EMSA. GST-HuR truncated domains were purified and eluted, and then, RNA-EMSA was performed using the ARE repeat and Cxcl2-ARE RNA (**b**) as the substrate. (**e–g**) PARP1 activation promotes HuR oligomerization along the target RNA. GST-HuR or GST (**e**), as well as D226 mutants (**f**, **g**) were purified and eluted, either subjected to PARylation or not, and then incubated with the biotin-labeled tandem ARE repeat RNA oligos as indicated (**b**). The gel retardation assay was performed to detect the binding of proteins to the probe

Next, we examined the roles of RNA in HuR–HuR interactions and the effects of PARylation in vitro. GST-HuR was either PARylated or not, and then incubated with His-HuR in the presence of an increasing amount of ARE-containing RNA oligos. Then, the amount of GST-HuR pulled-down by His-HuR was examined using western blotting. The presence of the RNA substrate increased the interactions between GST-HuR and His-HuR in a dose-dependent manner, and the PARylation of GST-HuR enhanced its interaction with His-HuR (Fig. 4a). However, when the GST-HuR D226A mutant was applied, incubation with PARP1 or the RNA substrate did not produce the effects shown by wild-type (WT) GST-HuR. Thus, while D226 PARylation provides a favorable conformation for HuR interactions, the presence of cellular RNA may further stabilize the complex.

However, we speculated that the formation of the HuR oligomeric complex gives rise to the occupation of multiple copies of HuR along the ARE-containing substrate. To test this hypothesis, we first detected the domain(s) of HuR that were majorly involved in RNA association. RNA-EMSA was performed using HuR domain mutants. ARE-rich and Cxcl2-ARE RNA probes, which had been utilized in our previous study [14], were used as the substrates (Fig. 4b). RRM1, but not other domains, effectively formed retarding complexes (Fig. 4c). An eight to nine nucleotide HuR-binding site appears to exist in the cooperative oligomeric complex [39]. To rule out multiple copies of HuR being harbored on the tandem ARE-containing probes, we also used a Cxcl2-ARE probe that contains only one HuR-binding site to perform an RNA-EMSA assay. Full-length HuR elicited multiple shifted bands, reflecting the association of oligomeric HuR with both substrates, whereas mutants RRM1 and RRM1 + RRM2 did not, and the mutant lacking RRM3 but containing HNS formed weak retarding complexes (Fig. 4d). When HuR was unable to interact with itself, even the association of monomeric HuR with the substrate was impaired, supporting the hypothesis that HuR oligomerization favors substrate association (Fig. 4d).

To further understand PARylation’s effects on the oligomerization of HuR along the target RNA, GST-HuR was either subjected to PARylation or not, and then an RNA-EMSA assay was performed. PARylation markedly enhanced the binding of both monomeric and oligomeric HuR with the ARE-containing probes, and this was weakened by the PARP inhibitor Ola (Fig. 4e). The result raise an issue that whether D226 is also crucial for substrate association. To this end, an RNA-EMSA with the D226A mutant along with increasing levels of ARE-probe in comparison to WT HuR was performed. The results showed that the bands containing monomeric WT HuR and D226A mutant had no significant difference. However, it’s interesting to note that binding of oligomeric HuR with the ARE-containing probes was weakened by the D226 mutation to some extent (Fig. 4f), which reflects the importance of aspartic acid for the conformation of HNS and RRM3 domains. Next, GST-HuR and the GST-HuR D226A were subjected to PARylation followed by incubation with increasing levels of ARE-probe. The formation of bands containing both monomeric and oligomeric HuR was significantly reduced by D226 mutation (Fig. 4g), in line with the result shown by Fig. 4e. Collectively, the data suggested that HuR’s substrate association and oligomerization are mutually beneficial, and importantly, this reciprocity is a functional subsequence of HuR PARylation.

### PARP1 improves HuR’s function through the inhibition of miRISC-mediated RNA cleavage

**Fig. 5:**
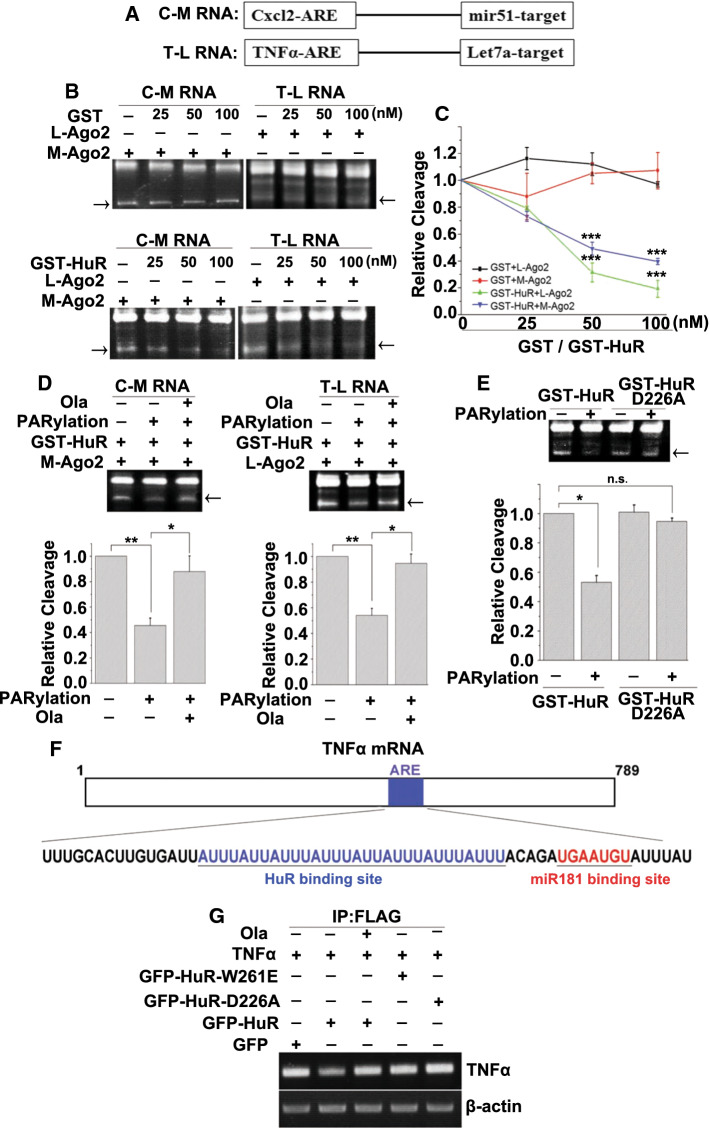
PARP1 improves HuR’s function in inhibiting RNA cleavage by miRISC. **a** Schemes of target RNAs used for the cleavage assay. C-M RNA, RNA probe containing Cxcl2 ARE and the mir51-binding site. T-L RNA, RNA probe containing TNFα ARE and the let7a-binding site. **b**, **c** Representative in vitro cleavage reactions performed with indicated target RNAs and increasing GST or GST-HuR concentrations. The cleavage products are indicated by arrows (**b**). PhosphorImaging quantification of cleavage reactions similar to those shown in panels B (**c**). ***p < 0.001. **d**, **e** PARP1 improves HuR’s function in inhibiting miRISC-mediated RNA cleavage. An in vitro PARylation assay was performed using purified GST-HuR (**d**) as well as D226 mutants (**e**) in the presence or absence of recombinant PARP1 enzyme. Then, PARylated HuR (**d**) and D226 mutants (**e**) were added to the in vitro cleavage reactions. The cleavage products are indicated by arrows. PhosphorImaging quantification of cleavage reactions similar to those shown in panels B (lower). **p < 0.01, *p < 0.05, n.s. = not significant. **f** Schemes of human TNFα mRNA 3′UTR having HuR and potential miR-181 sites. **g** PARylation increases the competition of HuR with miRISC in vivo. Endogenous HuR in HEK293 cells was silenced using siRNA targeting a distinct sequence of the human HuR, and then, GFP, GFP-mHuR, GFP-mHuR W261E or GFP-mHuR D226A together with Flag-Ago2 expression plasmids were transfected. Cells then stimulated with TNFα (± Ola) for 1 h, and then RNA-IP was conducted using a FLAG antibody. The bead–antibody–protein/mRNA complexes were subjected to PCR to detect pulled-down TNFα mRNA levels

The oligomerization of HuR attenuates miRNA-mediated target RNA decay by promoting miRISC dissociation [16]. miRNAs function in the form of ribonucleoprotein particles, miRNPs (or miRISCs) [42]. Argonaute (Ago) proteins are the best characterized essential components of miRISC. Among the Ago proteins, only Ago2 is catalytically competent to endonucleolytically cleave target RNA [43]. To investigate whether PARylation increases HuR oligomerization and alleviates miRNA-mediated RNA degradation, we performed an miRISC-mediated cleavage assay. The miRNA-enriched miRISC was prepared by the co-expression of human Flag-tagged Ago2 and the mir51 primary precursor (pri-miRNA) or let-7a miRNA in HEK293 cells (Figure S3A), followed by affinity purification using anti-FLAG Ab beads. The expression of tagged Ago2 was verified by western blotting (Figure S3B). Then, we determined whether the addition of purified recombinant HuR affects the miRISC-mediated cleavage of RNA containing the mir51 or let-7a sequences and the HuR-binding site (Cxcl2-ARE or TNFα-ARE) as described previously [16] (Fig. 5a). The target RNA was specifically cleaved by the miRISC (Figure S3C). The addition of GST did not affect the cleavage of the target mRNA by miRISC, but a gradual increase in GST-HuR resulted in a dose-dependent repression of substrate cleavage (Fig. 5b, c). Furthermore, whether the PARylation of HuR enhances its inhibition of RNA cleavage was investigated. Purified and eluted GST-HuR and the D226A mutant were either subjected to PARylation or not, followed by incubation with the target RNA and miRISC complex. HuR dramatically inhibited miRISC-mediated cleavage of the target mRNA. Incubation with PARP1 enhanced the inhibitory effects of HuR, which were reversed by the addition of Ola (Fig. 5d). After PARP1 incubation, the HuR D226A mutant did not block the miRISC-mediated cleavage of the target mRNA (Fig. 5e).

To further clarify the role of HuR PARylation in the miRISC-mediated cleavage of the target mRNA *in cellulo*, GFP-tagged WT murine HuR (mHuR; which is 100% homologous with human HuR at amino acid level) and W261E and D226A mutants, together with Flag-Ago2, were transfected into endogenous HuR-silenced HEK293 cells (Figure S3D). They were then exposed to TNFα with or without Ola. Flag-Ago-precipitated mRNAs from differently-treated cells were analyzed. The TNFα mRNA 3′UTR containing HuR and miR181-binding sites was used as a target [44] (Fig. 5f). The RNA-immunoprecipitation (RNA-IP) revealed that TNFα exposure markedly blocked Ago’s association with TNFα mRNA in WT mHuR-expressing cells, and this was reversed by the addition of Ola. However, the expression of GFP, GFP-mHuR W261E and GFP-mHuR D226A did not perturb the association of Ago with the target (Fig. 5g). Thus, the PARylation of HuR appears to promote its oligomerization along the RNA substrate, which may competitively remove the miRISC, thereby inhibiting target RNA cleavage.

### PARylation is required for the enhancement of mRNA stabilization mediated by HuR oligomerization in response to inflammatory stimulation

**Fig. 6:**
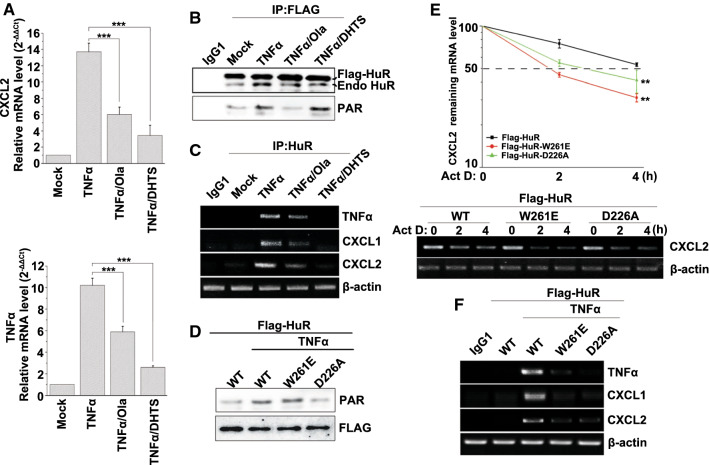
PARylation enhances the sequential oligomerization of HuR and increased mRNA stability. **a** Inflammation-related mRNA expression is impaired in HuR oligomerization-disrupted cells. HEK293 cells were treated with TNFα together with Ola or DHTS. Then, real-time PCR was performed to detect the mRNA expression levels of CXCL2 and TNFα. ***p < 0.001. **b** Ola and the HuR-specific regulator DHTS disrupt HuR self-interactions. HEK293 cells were transfected with Flag-HuR, and then, challenged with TNFα together with Ola or DHTS. Immunoprecipitates were prepared using control IgG1 or an anti-FLAG antibody and then, subjected to western blotting to detect the interactions of Flag-HuR with endogenous HuR and the PARylation of Flag-HuR. Endo HuR, endogenous HuR. **c** Ola and the HuR-specific regulator DHTS inhibited the binding of HuR to pro-inflammatory gene mRNAs. HEK293 cells were treated with TNFα together with Ola or DHTS. Then, RNA-IP was conducted using control IgG1 or an anti-HuR antibody. The bead–antibody–protein/mRNA complexes were subjected to PCR to detect pulled-down TNFα, CXCL1 and CXCL2 mRNA levels. **d** The W261 mutation does not impair the PARylation of HuR in response to TNFα exposure. HEK 293 cells were transfected with wild-type (WT) Flag-HuR, W261E and D226A mutant plasmids respectively, and then either challenged with TNFα or not for 1 h. Immunoprecipitates were prepared using an anti-FLAG antibody and then subjected to western blotting to detect the PARylation of Flag-HuR. **e**, **f** W261E and D226A, mutants that do not undergo oligomerization and PARylation, failed to enhance the stability of inflammation-related mRNAs. Endogenous HuR in HEK293 cells was silenced using siRNA targeting a distinct sequence of the human HuR, and then, WT mHuR, W261E and D226A mHuR expression plasmids were transfected. Cells were stimulated with TNFα for 0.5 h to boost inflammatory gene expression levels and then, they were subjected to transcriptional inhibition for different lengths of time (as indicated). Real-time PCR (upper) and RT-PCR (lower) were performed to assess the remaining CXCL2 mRNA levels. Half-lives of different samples are indicated in the inset. A two-way analysis of variance indicated the significance between the Flag-HuR-W261E/Flag-HuR and Flag-HuR-D226A/Flag-HuR groups at **p < 0.01 (**e**). HEK293 cells were transfected with WT Flag-HuR, as well as W261E and D226A mutant plasmids. RNA-IP was conducted using control IgG1 or an anti-FLAG antibody following the procedure described above (**c**), and the levels of precipitated TNFα, CXCL1 and CXCL2 mRNAs were detected by RT-PCR and electrophoresis (**f**)

Although HuR oligomerization has been observed, its impact on target mRNAs in cells under stress condition and the implication of PARylation remain uncertain. To increase our understanding, a tanshinone group compound, 15,16-dihydrotanshinone-I (DHTS), was utilized to disrupt HuR oligomerization [34]. We treated cells with TNFα together with Ola or DHTS. Real-time PCR showed that increases in the levels of CXCL2 and TNFα mRNAs in TNFα-treated cells were attenuated by the addition of Ola or DHTS (Fig. 6a). Flag-HuR was also transfected into cells to test the effects of PARylation as well as oligomerization on HuR’s function. Although the treatment of DHTS did not affect the PARylation of Flag-HuR, TNFα treatment-induced interactions of Flag-HuR with endogenous HuR were markedly reduced by Ola or DHTS (Fig. 6b and Figure S4A). Furthermore, RNA-IP assays were performed to detect the binding of HuR with the target mRNAs. TNFα exposure markedly promoted the association of HuR with the target mRNAs (CXCL1, CXCL2 and TNFα), and this was significantly reduced by the addition of Ola or DHTS (Fig. 6c). Thus, once the oligomerization of HuR in cells is disrupted, the stabilized regulation of mRNA by HuR may be inhibited.

Moreover, Flag-tagged WT HuR as well as W261E and D226A mutants were transfected into HEK293 cells respectively, and then, an IP assay was performed to detect the PARylation of HuR and the mutants. The results showed that W261E mutation did not impair the PARylation of HuR under inflammatory stimulation (Fig. 6d). We also performed in vitro PARylation of GST-HuR and the W261E mutant, followed by pull-down assay using His-HuR. The results showed that even if the mutant was still capable to undergo PARylation, the dimerization of HuR was significantly inhibited by W261E mutation (Figure S4B). Then, Flag-tagged WT mHuR and the mutants were transfected independently into endogenous HuR-silenced HEK293 cells and then exposed to TNFα. Further investigations into the stability of the pro-inflammatory gene’s mRNA showed that the half-life of the remaining CXCL2 mRNA in WT mHuR-expressing cells was ~ 4 h, and this was reduced to ~ 2 h in D226A or W261E mHuR-expressing cells (Fig. 6e). In addition, an RNA-IP assay was performed. As expected, the W261E and D226A mutants significantly inhibited Flag-HuR binding with target mRNA (Fig. 6f). The data indicated that the oligomerization is crucial for HuR’s role in mRNA stability, and PARP1 influence HuR’s function by promoting the sequential oligomerization of the latter.

### PARP1 enhances HuR oligomerization and promotes an inflammatory reaction in mouse lungs

**Fig. 7:**
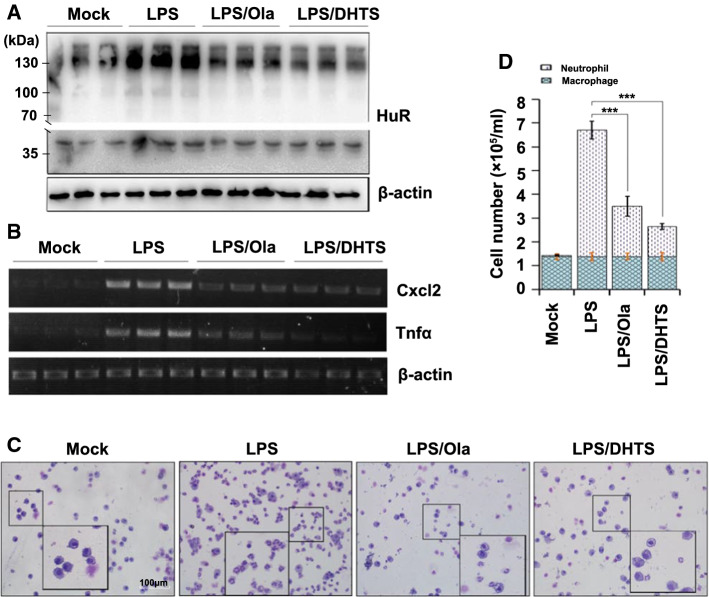
PARP1 regulates inflammatory gene expression by increasing HuR oligomerization in mouse lungs. **a**, **b** The activation of PARP1 is attributed to the oligomerization of HuR in mouse lungs. Mouse lungs were challenged with LPS (1 mg/kg) for 1 h, with or without an Ola (10 mg/kg) or DHTS (5 mg/kg) intraperitoneal pretreatment at 30 min prior to the LPS challenge. Lung homogenates were prepared, and after incubating either with or without DSS, the lung homogenates were subjected to western blotting using an anti-HuR antibody (**a**). The levels of Cxcl2 and Tnfα mRNAs in mouse lungs were detected by RT-PCR and electrophoresis (**b**). **c**, **d** Visual depiction and quantification of cells in bronchoalveolar lavage fluids (BALFs) from differently treated mice. Mice were mock treated or challenged with LPS in the presence or absence of an Ola or DHTS pretreatment. After 16 h, mice were euthanized, lungs were lavaged, and the cell numbers in BALFs were determined (**c**). Differential cell counts were performed after modified Wright-Giemsa staining, *n* ≥ 5 (**d**). Scale bar, 100 μm. ***p < 0.001

To investigate the effects of PARP1 on HuR oligomerization, as well as the consequences in an in vivo scenario, mouse lungs were exposed to LPS through an intranasal route with or without an intraperitoneal pretreatment with Ola or DHTS. LPS induced a notable increase in the level of HuR oligomerization in mouse lungs, and this was blocked by a pretreatment with Ola or DHTS (Fig. 7a). Additionally, the pro-inflammatory gene’s mRNA level markedly increased after 1 h of LPS exposure, and this was also significantly reduced by an Ola or DHTS treatment (Fig. 7b). Mice were euthanized 16 h after the LPS challenge, the lungs were lavaged and the cell numbers in the bronchoalveolar lavage fluid were determined. Challenges with LPS induced a robust recruitment of neutrophils to the airways, and this was markedly reduced in samples from Ola and DHTS-treated animals (Fig. 7c, d). The results implied a potential physio-pathological impact of HuR oligomerization and an important regulatory role of PARP1 in response to inflammatory stimuli.

## Discussion

A large number of studies have addressed the involvement of PARP1 in gene expression by modulating transcriptional activation and elongation [28, 45]. Additionally, some RBPs that are modified by PARylation have recently been found to participate in RNA processing, including splicing, polyadenylation and mRNA turnover [14, 46]. A new perspective on PARP biology that involves mRNA metabolism is emerging [27, 47]. Our previous study illustrated a novel role of PARP1 in the HuR-mediated mRNA stabilization of pro-inflammatory genes [14]. Here, we further dissected the precise molecular mechanism, demonstrating that in response to inflammatory stimuli, HuR’s PARylation at D226 promotes its oligomerization along the target mRNA, thereby preventing miRISC-mediated mRNA cleavage.

Although the oligomerization/multimerization of HuR and related family members, such as HuB, HuD and Drosophila ELAVL, along with RNA substrates, have been previously shown using recombinant proteins or in neural cells [33, 34, 39, 48, 49], our present study provided evidence of stimulation-dependent HuR self-interactions and the associated regulatory mechanism. The in situ chemical crosslinking analysis revealed bands at ~ 70, 100 and 130 kDa, which may be the dimeric, trimer and tetrameric forms of HuR, respectively, and the PLA analysis further verified the direct self-binding of HuR (Fig. 1). Importantly, the enhancement of HuR oligomerization induced by TNFα exposure was diminished when PARP1 inhibition or depletion occurred. Moreover, HuR D226A, which cannot be PARylated, was also unable to undergo oligomerization in vitro and in vivo (Fig. 1).

HuR is composed of three highly conserved canonical RRMs. RRM1 and RRM2 are positioned next to each other, followed by a hinged HNS region and then the C-terminal RRM3. HNS and RRM3 have been shown to mediate the oligomerization/multimerization of HuR and *Drosophila* ELAVL [39, 41, 48]. The conserved tryptophan in the acidic α1 helix of RRM3, W261 in HuR and W419 in ELAVL, are essential for RRM3 interactions. Moreover, the last third of HNS is indispensable for ELAVL multimerization [41]. Intriguingly, although the HNS region is less conserved among ELAVL/Hu family members, an aspartic acid located at the boundary between the second and the last thirds of HNS (D226 in HuR) is highly conserved, implying its functional importance.

**Fig. 8:**
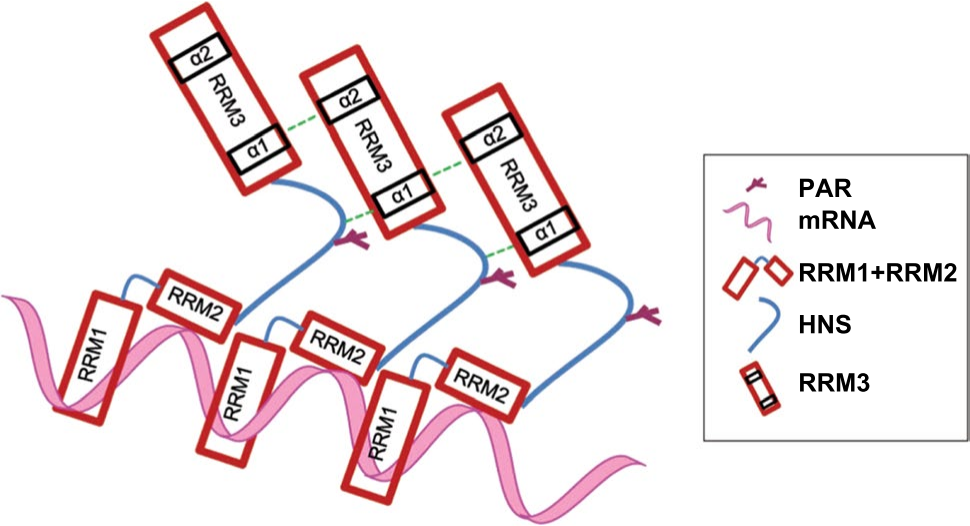
Potential models of D226 PARylation-enhanced HuR oligomerization

While a structural investigation of the full-length HuR remains challenging owing to its poor stability and solubility, studies of the separate RRMs have enabled us to obtain functional insights. While HuR’s N-terminal RRM1 and RRM2 domains are fixed and persistently oriented, HuR’s C-terminal RRM3 and HNS modules seem to be unoriented [36]. We deduced that, in the resting state, the acidic α helices, which mediate RRM3 interactions, dynamically associate with the basic NLS in the second third of the HNS region. PARylation at D226 introduces a bulky negatively charged PAR polymer that is repulsive to acidic α helices, thus allowing the last third of HNS and α helices of the RRM3 to form a favorable interface for ELAVL/Hu family member interactions (Fig. 8). W261, which is located in the first α helix of RRM3, is crucial for ELAVL/Hu protein interactions, and structural simulations have provided a view into how W261 sites from two HuR molecules interact with each other [36, 37]. However, the question of how W261 interactions accommodate HuR oligomerization/multimerization remains unanswered. We hypothesize that the parallel and interlaced interactions of HNS and RRM3 domains may supply the answer and that the PARylation of D226 may favor this interface layout (Fig. 2 and 8). To support this speculation, intensive investigations are required.

All three RRMs interact with RNA ligands [36, 37, 50], and the data [51, 52] indicate a high affinity of RRM1 for AU-rich substrates (Fig. 4). The optimal HuR’s RNA-binding involves all three RRMs, and this also supports the speculation that HuR oligomerization through HNS and RRM3 favors its binding with target mRNAs. D226 is a target of caspases (e.g., caspase-3 and -7) [26] under lethal stress conditions [26, 38], which adds an additional layer of subtleness to the regulation of HuR functions. Cleavage of HuR at D226 produces a HNS-RRM3 fragment, which may competitively inhibit HuR oligomerization/multimerization and subsequently the association with the mRNA targets. D226 is a site recognized by both PARP1 and caspases, signifying its decisive fate-related effects. Thus, in response to different external signals or levels of external stresses, actions on D226 determine whether the cell undergoes apoptosis or an inflammatory response.

HuR is predominantly localized in the nucleus, but its effects on mRNA stability and translation are linked to its transport to the cytoplasm, which requires the HNS domain of HuR and transport proteins, such as chromosome region maintenance 1 or transporting 2 [12, 53–55]. HuR forms multimers in in the cytoplasmic fractions of glioma cells [34]. However, here, we demonstrate that the oligomerization of HuR occurs both in the cytoplasm and nucleus, and it is significantly enhanced under stress conditions. This implies that HuR may initially bind mRNAs in the nucleus and accompany them into the cytoplasm, providing ongoing protection from the degradation machinery (Fig. 3). In addition to its function in the regulation of mRNA stability, HuR also participates in pre-mRNA splicing in the nucleus [56–59]. Although there is no direct intracellular evidence, previous in vitro studies showed that ELAVL multimerizes on the *erect wing* (*ewg*)-binding site and forms a dodecameric complex of 135 nucleotides in the last ewg intron [48]. Here, HuR underwent a low level of oligomerization in the nucleus even in the absence of stimulation, which implied that, in addition to regulating the stability of mature mRNA, oligomeric HuR may also be involved in alternative splicing. Furthermore, PARP1, as an important nuclear protein, was recently shown to bind to pre-mRNA and regulate the alternative splicing process [30, 60–62]. Thus, the influence of PARylation on the oligomerization of HuR may have other profound effects in different RNA metabolism pathways, and this needs to be explored in the future.

HuR protein oligomerzation/multimerization has been documented [33, 34, 36, 37, 63], but how the molecular mechanism responsible for HuR oligomerzation is regulated in vivo has not been addressed. Here, once the activity of PARP1 was inhibited or the site of HuR PARylation was mutated, the oligomerization of HuR was markedly inhibited and the stability of the pro-inflammatory factors was significantly reduced (Figs. 6 and  7). Importantly, the significant impairment of HuR-W261E and -D226A binding to several cytokine mRNA targets in human cells was observed. The reduced levels of those mRNAs in cells expressing HuR W261E and D226A presumably resulted from their defective stabilization capabilities, which were caused by less efficient HuR binding (Fig. 6).

However, although HuR regulates the stability or translation of several mRNAs, the associated molecular mechanisms remain elusive. Over 75% of mRNAs having Ago-binding sites in their 3′UTRs also contain HuR-binding sites, suggesting a combinatorial regulation of mRNA stability by HuR and miRNAs [64]. A whole-transcript expression profiling study showed a significant enrichment in overlapping/adjacent HuRs and Ago-binding sites in the 3′UTRs of targeted mRNAs. The transcript classes having HuR-binding sites were destabilized upon HuR knockdown regardless of whether the miRNA sites overlapped or were adjacent to the HuR [65]. Here, using an in vitro mRNA decay assay, we showed that HuR interferes with miRISC activity even when the miRISC site is located outside of the 20-bp HuR-binding region, suggesting that the HuR effect is unlikely to be caused by the direct spatial blockade of the miRISC site. The above studies all indicate that HuR relieves miRNA-mediated repression, most likely owing to the oligomerization of the HuR protein, to occupy overlapping or adjacent miRNA-binding sites. Furthermore, here, in vitro and in vivo data suggested that the PARylation of HuR increased the ability of HuR to compete with miRISC (Fig. 5). PARylation may alter the conformational characteristics of the HuR protein, which likely promotes the exposure of the interface for oligomerization and increases the chance of oligomeric HuR binding along the target RNA, which in turn attenuates miRISC’s mRNA cleavage (Fig. 5).

The oligomerization of HuR increased in LPS-treated mouse lungs, and this effect was significantly reduced by an Ola or DHTS treatment. In addition, increases in the levels of pro-inflammatory gene mRNAs, as well as the recruitment of neutrophils, in airways induced by LPS were diminished by PARP1 inhibition or DHTS (Fig. 7). These data further indicate a crucial role for PARP1 in HuR oligomerization in a physiopathological context. Notably, HuR is associated with tumorigenesis through the promotion of expression levels of proteins that increase proliferation, enhance cell survival and facilitate invasion and metastasis [66]. Additionally, PARP1 is overexpressed in a variety of cancers, including glioblastoma, prostate and breast cancers [67–70]. This positive correlation between the expression levels of HuR and PARP1 in tumor cells may indicate the significance of PARP1 in HuR functions in another physiopathological context. PARP1 modifies HuR, which at least partly, accounts for the elevated HuR oligomerization/multimerization observed in tumor cells [34, 71], thereby promoting the stabilization of tumor-associated factor mRNAs.

Overall, the formation of HuR oligomers is a complex process that may be affected by intracellular conditions (such as reactive oxygen species, pH and stress levels), cofactors and post-translational modifications [34]. Here, we showed the important role of PARP1 in the regulation of HuR oligomerization. In quiescent cells, the HuR protein mostly exists in monomeric form, and the ARE-containing mRNAs are rapidly destabilized by miRISCs or other destabilizing RBPs. During immune responses, PARP1 promotes the oligomerization of HuR through PARylation, and HuR oligomerization gives HuR it a decisive advantage in stabilizing mRNA, which in turn competes with miRISC and plays a role in the regulation of mRNA. HuR oligomerization may contribute to the storage of ARE-containing mRNAs. This enhancement of HuR oligomerization, which is affected by PARP1, can be thought of as an adaptive survival mechanism for cells under stressful conditions. The PARylation of HuR promotes the oligomerization of HuR, thereby mitigating miRNA repression from a distance, representing a new mechanism of mRNA regulation.

## Electronic supplementary material

Below is the link to the electronic supplementary material.

Supplementary material 1 (DOCX 19 kb)

Supplementary material 2 (PDF 24292 kb)

Supplementary material 3 (PDF 41439 kb)

Supplementary material 4 (PDF 11363 kb)

Supplementary material 5 (PDF 1932 kb)

## Abbreviations

AREs: AU-rich elements
3′ UTRs: 3′ Untranslated regions
RBPs: RNA-binding proteins (RBPs)
HNS: Nucleocytoplasmic shuttling sequence
ELAVL: Embryonic lethal abnormal vision-like
PARylation: Poly ADP-ribosylation
PAR: Poly ADP-ribose
miRISC: MiRNA-induced silencing complex
HEK293: Human Embryonic Kidney cells
Ola: Olaparib
Act D: Actinomycin D
DHTS: Tanshinone group compound 15,16-dihydrotanshinone-I
DSS: Disuccinimidyl suberate
PLA: Proximity ligation assay
WT: Wild-type
CEs: Cytosolic extracts
NEs: Nuclear extracts
LPS: Lipopolysaccharide
Ago: Argonaute
