# Supplementary material for: Poly(ADP-ribosyl)ation enhances HuR oligomerization and contributes to pro-inflammatory gene mRNA stabilization
Source: Cell Mol Life Sci. 2020 Aug 13;78(4):1817–35. doi: 10.1007/s00018-020-03618-4 (PMC7904744; doi:10.1007/s00018-020-03618-4)
Supplement: Supplementary file 1 — Supplementary material 1 (DOCX 19 kb) [file 18_2020_3618_MOESM1_ESM.docx]

**Supplemental Information**

**Table S1.** DNA oligonucleotides used for construction of templates for *in vitro* transcription

| T7: | TTAATACGACTCACTATAGGG |
| --- | --- |
| C-M DNA Antisense (containing Cxcl2 ARE and mir51 binding site): | TGTAACGTTACCCGTAGCTCTGACTCTCTCGTACAGCTACACATAGATAAATAAATAACCCCTATAGTGAGTCGTATTAA |
| T-L DNA Antisense (containing TNFα ARE and let7a binding site): | TGTAACGTAGGTAGTAGGTTGTATAGTCTGACTCTCTCGTACAGCTACACATAGATAAATAAATAACCCCTATAGTGAGTCGTATTAA |

**Table S2.** Sequences of target RNAs.

| C-M RNA (containing Cxcl2 ARE and mir51 binding site): | GGUUAUUUAUUUAUCUAUGUGUAGCUGUACGAGAGAGUCAGAGCUACGGGUAACGUUACA |
| --- | --- |
| T-L RNA (containing TNFα ARE and let7a binding site): | GGUUAUUUAUUUAUCUAUGUGUAGCUGUACGAGAGAGUCAGACUAUACAACCUACUACCUACGUUACA |

**Table S3.** An amino-acid sequence of HNS and RRM3 from HuR

| The first one third of HNS | F:CCCAACCAGAACAAAAACGTGGCACTCCTCTCGCAGCTGTACCACTCGCCAGCG  R:CGCTGGCGAGTGGTACAGCTGCGAGAGGAGTGCCACGTTTTTGTTCTGGTTGGG |
| --- | --- |
| The last one third of HNS | F:GTCGATCACATGAGCGGGCTCTCTGGCGTCAACGTGCCAGGAAACGCCTCCTCCGGCTGG  R:CCAGCCGGAGGAGGCGTTTCCTGGCACGTTGACGCCAGAGAGCCCGCTCATGTGATCGAC |
| His-HuR-RRM3-α1 | F:GGGCAGGATGCCGACGAGGGGATCCTCTGGCAGATGTTTGGGCCGTTTGGT  R:ACCAAACGGCCCAAACATCTGCCAGAGGATCCCCTCGTCGGCATCCTGCCC |
| GST-HuR-RRM3-α2 | F:ATGACAAACTATGAAGAAGCCGCGATGGCCATAGCCAGCCTGAACGGCTACCGCCTGGGG  R:CCCCAGGCGGTAGCCGTTCAGGCTGGCTATGGCCATCGCGGCTTCTTCATAGTTTGTCAT |
| GST-HuR-RRM3-β4 | F: TTACAGGTTTCCTTCAAAACC  R: GGTTTTGAAGGAAACCTGTAA |

**Supplemental legends**

**Supplemental Figure 1 - Inflammatory stimulation induces HuR oligomerization *in cellulo*.**

**(A)** The oligomerization of HuR shown in Figure 1A in the main text was quantified by analysis of band densitometry using the ImageJ software. ***p < 0.001 ; n＝3.

**(B)** The amount of co-precipitated endogenous HuR shown in Figure 1B in the main text was quantified by analysis of band densitometry using the ImageJ software. ***p < 0.001 ; n＝3. Endo HuR, endogenous HuR.

**(C)** Inflammatory stimulation induces HuR forming oligomers. GFP-HuR together with Flag-HuR was transfected into cells, and the lysates from differently treated cells were immunoprecipitated using an anti-FLAG antibody, followed by western blotting with the HuR antibody. The amount of co-precipitated endogenous HuR and GFP-HuR was quantified by analysis of band densitometry using the ImageJ software (lower). ***p < 0.001; n＝3. Endo HuR, endogenous HuR.

**(D)** PLA analyses of the oligomerization of HuR. HA-HuR together with Flag-HuR plasmids was transfected into cells, and then the cells were subjected to in vivo PLA assay with indicated antibodies. Scale bar, 10 μm.

**(E)** Activation of PARP1 enhances HuR self-association. GFP-HuR-transfected cells were mock-treated or TNFα-exposed (± Ola) for 1 h. Whole-cell extracts (WE) were prepared and immunoprecipitates were obtained using antibodies recognizing GFP. The association of GFP-HuR with endogenous HuR was detected by western blotting. The amount of co-precipitated endogenous HuR was quantified by analysis of band densitometry using the ImageJ software (right panel). ***p < 0.001; n＝3.

**(F, G)** The amount of co-precipitated endogenous HuR shown in Figure 1E (F) and 1F (G) in the main text was quantified by analysis of band densitometry using the ImageJ software. ***p < 0.001; n＝3. Endo HuR, endogenous HuR.

**Supplemental Figure 2 - PARP1 activity enhances HuR oligomerization.**

**(A)** HuR and PARP1 did not undergo caspase-mediated cleavage under inflammatory conditions. HEK 293 cells were treated with TNFα for 1 h. Whole-cell extracts were prepared, and then, the titrated samples were subjected to western blotting with HuR and PARP1 antibodies.

**(B, C)** HuR oligomerization *in vitro*. GST-HuR and His-HuR interact with each other. Different concentrations of GST and GST-HuR or His-HuR beads were incubated with the same amounts of His-HuR or GST and GST-HuR protein, and then, they were subjected to western blotting using an anti-His or GST antibody, respectively.

**(D)** The amount of His-HuR shown in Figure 2D in the main text was quantified by analysis of band densitometry using the ImageJ software. ***p < 0.001; n＝3.

**(E)** PARylation increased the oligomerization of HuR. An *in vitro* PARylation assay was performed using purified GST-HuR in the presence or absence of recombinant PARP1 enzyme with PJ34 or PARG. Then, the PARylated GST-HuR was incubated with His-HuR and subjected to western blotting with the His antibody. The amount of His-HuR was quantified by analysis of band densitometry using the ImageJ software. ***p < 0.001; n＝3.

**(F)** The amount of His-HuR shown in Figure 2E in the main text was quantified by analysis of band densitometry using the ImageJ software. ***p < 0.001; n＝3.

**(G)** The Illustration of different fragments of HuR.

**(H)** The amount of GST-HuR shown in Figure 2H in the main text was quantified by analysis of band densitometry using the ImageJ software. ***p < 0.001; n＝3.

**Supplemental Figure 3 - miRISC specifically cleaves target RNA bearing a sequence perfectly complementary to a mir51 primary precursor or let7a miRNA.**

**(A)** The Flag-Ago2 plasmid together with a synthesized mir51 primary precursor plasmid or let7a miRNA were transfected into cells, and immunofluorescence staining was conducted to detect the distribution of Flag-Ago and mir51 or let7a. Flag-Ago2, green. Mir51 primary precursor plasmid containing the RFP tag appears as red and let7a miRNA containing Cy3 also appears as red.

**(B)** Purification of miRISC. Flag-Ago2 together with a mir51 primary precursor plasmid or let7a miRNA were transfected into cells. Then, the cells extracts were purified using anti-FLAG antibody-coated beads, and this was followed by the elution of the miRISC using a FLAG peptide. The western blotting analysis showed the purification of mir51- and let7a-enriched Ago2 miRISC. Input, extract without transfection.

**(C)** Target RNA was specifically cleaved by the miRISC. Components added to cleavage reactions are indicated at the top. Cleavage products are indicated by arrows.

**(D)** GFP-tagged WT murine HuR, and W261E and D226A mutants together with Flag-Ago2 were transfected into endogenous HuR-silenced HEK293 cells, and the complexes were subjected to western blotting.

**Supplemental Figure 4 -** PARylation enhances the sequential oligomerization of HuR.

**(A)** The amount of co-precipitated endogenous HuR shown in Figure 6B in the main text was quantified by analysis of band densitometry using the ImageJ software. ***p < 0.001; n＝3. Endo HuR, endogenous HuR.

**(B)** An *in vitro* assay of the effect of PARylation on the oligomerization of HuR. An *in vitro* PARylation assay was performed using purified GST-HuR and W261E mutation in the presence or absence of recombinant PARP1 enzyme. Then, the PARylated GST-HuR and the mutant were incubated with His-HuR and subjected to western blotting with the His and PAR antibody.
