# Supplementary figures and images for: Poly(ADP-ribosyl)ation enhances HuR oligomerization and contributes to pro-inflammatory gene mRNA stabilization
Source: Cell Mol Life Sci. 2020 Aug 13;78(4):1817–35. doi: 10.1007/s00018-020-03618-4 (PMC7904744; doi:10.1007/s00018-020-03618-4)

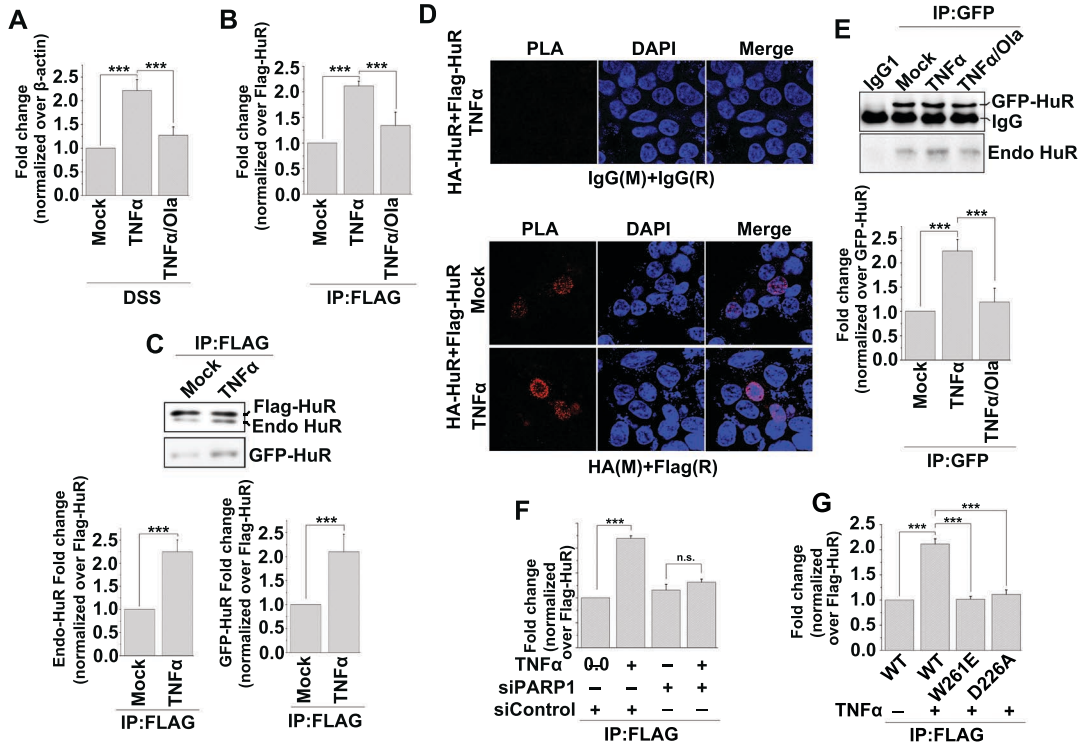

Supplement: Supplementary file 2 — Supplementary material 2 (PDF 24292 kb) [file 18_2020_3618_MOESM2_ESM.pdf]

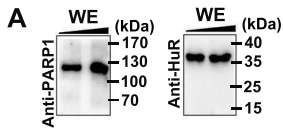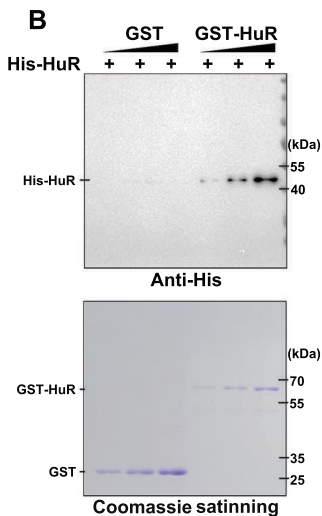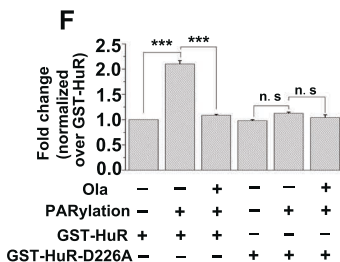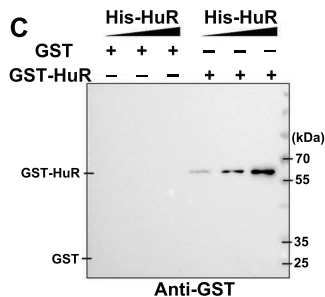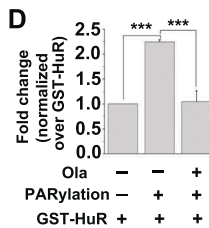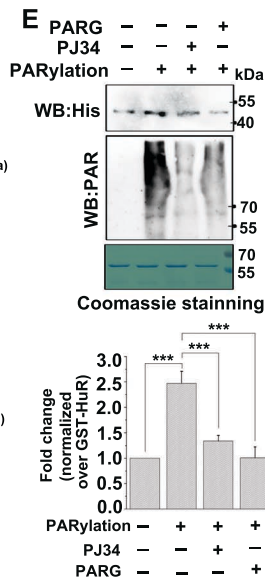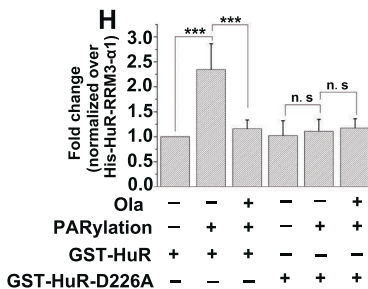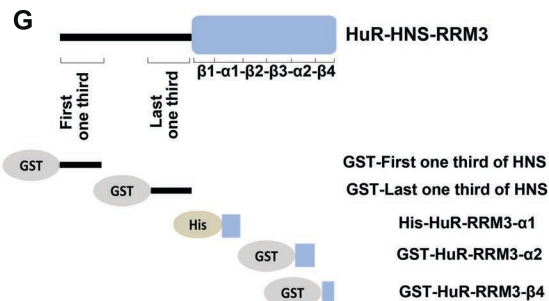

Supplement: Supplementary file 3 — Supplementary material 3 (PDF 41439 kb) [file 18_2020_3618_MOESM3_ESM.pdf]

**A**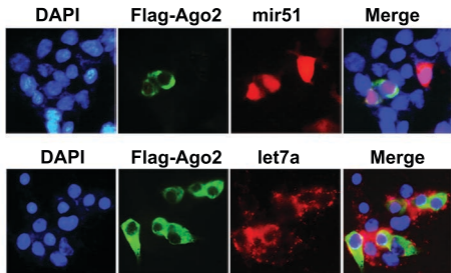

**B** mir51-enriched miRISC (M-Ago2)    let-7-enriched miRISC (L-Ago2)

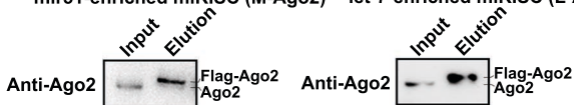**C**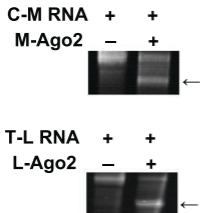**D**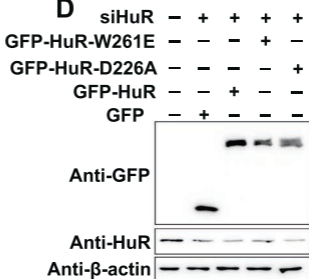

Supplement: Supplementary file 4 — Supplementary material 4 (PDF 11363 kb) [file 18_2020_3618_MOESM4_ESM.pdf]

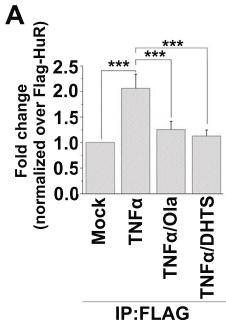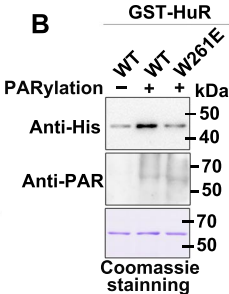

Supplement: Supplementary file 5 — Supplementary material 5 (PDF 1932 kb) [file 18_2020_3618_MOESM5_ESM.pdf]
